# HbA1c variability and diabetic peripheral neuropathy in type 2 diabetic patients

**DOI:** 10.1186/s12933-018-0693-0

**Published:** 2018-03-29

**Authors:** Jian-bin Su, Li-hua Zhao, Xiu-lin Zhang, Hong-li Cai, Hai-yan Huang, Feng Xu, Tong Chen, Xue-qin Wang

**Affiliations:** 1 Department of Endocrinology, The Second Affiliated Hospital of Nantong University and First People’s Hospital of Nantong City, No. 6, Haierxiang North Road, Nantong, 226001 China; 2 Department of Clinical Laboratory, The Second Affiliated Hospital of Nantong University and First People’s Hospital of Nantong City, No. 6, Haierxiang North Road, Nantong, 226001 China; 3 Department of Geriatrics, The Second Affiliated Hospital of Nantong University and First People’s Hospital of Nantong City, No. 6 North Haierxiang Road, Nantong, 226001 China

**Keywords:** Type 2 diabetes, Glycemic variability, HbA1c, Coefficient of variation, Diabetic peripheral neuropathy

## Abstract

**Background:**

Diabetic complications may be associated with impaired time-dependent glycemic control. Therefore, long-term glycemic variability, assessed by variations in haemoglobin A1c (HbA1c), may be a potential risk factor for microvascular complications, such as diabetic peripheral neuropathy (DPN). We investigated the association of HbA1c variability with DPN in patients with type 2 diabetes.

**Methods:**

In this cross-sectional study, 563 type 2 diabetic patients who had been screened for DPN and undergone quarterly HbA1c measurements during the year preceding enrolment were recruited. DPN was confirmed in patients displaying both clinical manifestations of neuropathy and abnormalities in a nerve conduction evaluation. HbA1c variability was assessed by the coefficient of variation of HbA1c (CV-HbA1c), and the mean of HbA1c (M-HbA1c) was calculated. In addition, medical history and clinical data were collected.

**Results:**

Among the recruited patients, 18.1% (n = 102) were found to have DPN, and these patients also presented with a higher CV-HbA1c than the patients without DPN (*p *< 0.001). The proportion of patients with DPN increased significantly from 6.9% in the first to 19.1% in the second and 28.5% in the third tertile of CV-HbA1c (*p* for trend < 0.001). After adjusting for initial HbA1c, M-HbA1c and other clinical factors via multiple logistic regression analysis, the odds ratios (ORs) for DPN in the second and third versus those in the first CV-HbA1c tertile were 3.61 (95% CI 1.62–8.04) and 6.48 (2.86–14.72), respectively. The area under the receiver operating characteristic (ROC) curve of CV-HbA1c was larger than that of M-HbA1c, at 0.711 (95% CI 0.659–0.763) and 0.662 (0.604–0.721), respectively. ROC analysis also revealed that the optimal cutoff value of CV-HbA1c to indicate DPN was 15.15%, and its corresponding sensitivity and specificity were 66.67% and 65.73%, respectively.

**Conclusions:**

Increased HbA1c variability is closely associated with DPN in type 2 diabetic patients and could be considered as a potent indicator for DPN in these patients.

## Background

Diabetic peripheral neuropathy (DPN), one of the most common microvascular complications, is closely connected to morbidity and mortality in type 2 diabetic patients [1]. DPN can facilitate the ulceration and gangrene of feet, which in turn increase the risk of non-traumatic amputation [1]. Furthermore, DPN is closely related to alterations in brain structure, especially a reduction in peripheral grey matter volume, which may be responsible for walking disabilities [2, 3]. Therefore, patients with DPN may be presented with an impaired quality of life and burdened with high costs of diabetes care [4].

The underlying pathogenesis of DPN is still under debate [5]. DPN is reported to be associated with glycemic exposure, the duration of diabetes, insulin resistance, visceral adiposity, dyslipidaemia, hypertension and so on [5]. However, evidence suggests that only tight glycemic control monitored by haemoglobin A1c (HbA1c) levels can ameliorate or prevent neuropathy [6]. Microvascular complications of diabetes may be associated with impaired time-dependent glycemic control [7]. In addition, glycemic variability is currently recognized as a marker of impaired glycemic control, which is a potential predictor for diabetic complications [8, 9]. Therefore, long-term glycemic variability assessed by HbA1c variability over the course of several months may be a reliable risk factor for microvascular complications, including diabetic neuropathy. Jun et al. [10] has demonstrated that HbA1c variability is significantly associated with the presence and severity of cardiovascular autonomic neuropathy (CAN) in type 2 diabetic patients. However, the role of HbA1c variability in DPN is not well known.

Therefore, we designed a study to estimate the association between the long-term glycemic variability assessed by HbA1c variability and DPN in type 2 diabetic patients.

## Methods

### Study design and participants

**Fig. 1:**
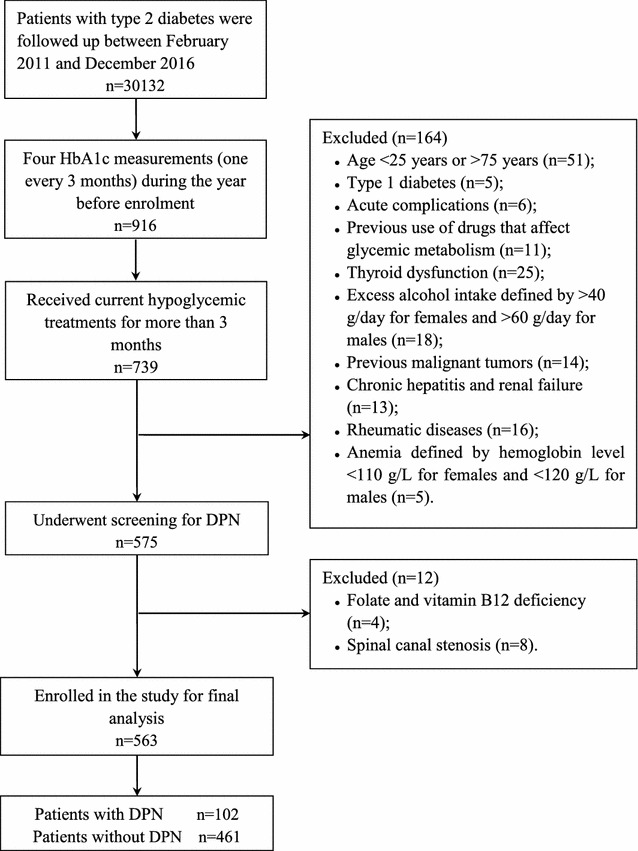
The study diagram

We performed a cross-sectional observational study of participants with type 2 diabetes who were followed up at the Second Affiliated Hospital of Nantong University between February 2011 and December 2016. Inclusion criteria were as follows: (1) type 2 diabetes diagnosed based on the statement of the American Diabetes Association (ADA) in 2011 [11]; (2) four HbA1c measurements (one every 3 months) over the year preceding enrolment; (3) 25–75 years old; (4) current hypoglycemic treatment for more than 3 months. Additionally, exclusion criteria included the following: (1) previous history of type 1 diabetes; (2) acute complications, i.e., ketoacidosis and hyperglycemic hyperosmolar status; (3) previous drugs uses that affect glycemic metabolism, i.e., steroids; (4) thyroid dysfunction; (5) excess alcohol intake defined by > 40 g/day for females and > 60 g/day for males; (6) previous malignant tumours; (7) chronic hepatitis and renal failure; (8) rheumatic diseases; (9) anaemia defined by a haemoglobin level < 110 g/L for females and < 120 g/L for males; (10) folate and vitamin B12 deficiency; (11) spinal canal stenosis. The study diagram is shown in Fig. 1. Finally, a total of 563 participants with type 2 diabetes were included in the study. The study procedures conformed to the guidelines of the Declaration of Helsinki, and each patient agreed to participate and signed the informed consent form. The study procedures were reviewed and approved by the medical research ethics committee of Second Affiliated Hospital of Nantong University.

### Basic data collection

During enrolment, medical histories were taken and routine physical examinations of the participants were performed by experienced physicians. Medical history included age, sex, past illness (i.e., hypertension, malignancy and spinal disease), personal behaviours (i.e., smoking and drinking), drug uses (i.e., antihypertensive agents and statins medications), current hypoglycemic treatments (i.e., lifestyle intervention, insulin injections, insulin secretagogues, metformin or thiazolidinediones use), and reported hypoglycemic events during the year preceding enrolment. Somatometric parameters were collected after physical examination, including body mass index, systolic blood pressure (BP), diastolic BP, etc. Hypertension was defined as a systolic BP ≥ 140 mmHg, a diastolic BP ≥ 90 mmHg or a history of hypertension or taking antihypertensive agents.

### Estimation of glycemic parameters

Four HbA1c values (one every 3 months) of the participants during the year preceding enrolment were obtained from the Hospital Information System. HbA1c variability was assessed by the coefficient of variation of HbA1c (CV-HbA1c). The initial HbA1c value was also documented for further analysis. In addition, long-term hyperglycaemia over the year preceding enrolment was assessed by the mean of HbA1c (M-HbA1c).

### DPN assessment

DPN screening was conducted by endocrinologists in a quiet and secluded room. Confirmed DPN was diagnosed in patients displaying both the presence of neurological symptoms/signs and abnormalities in nerve conduction evaluations [12]. Neuropathic symptoms included numbness, tingling, unsteadiness, prickling or burning pain in the legs and/or feet. Neuropathic signs were defined as reduced or absent ankle reflexes (using an appropriate reflex hammer) and reduced or absent distal sensation, including vibration perception (using a 128-Hz tuning fork), touch sensation (using a 10-g monofilament), thermal discrimination (using cold and warm objects), pinprick sensation (using a pin) and proprioception. Signs were evaluated through careful neurologic examinations. An evaluation of nerve conduction in each patient was performed using an electromyogram (MEB-9200K, Nihon Kohden). Nerve conduction of the common peroneal, posterior tibial and sural nerves was assessed on both sides. Nerve conduction abnormalities were identified when the electromyogram presented with at least one abnormal nerve parameter, including delayed latency, decreased amplitude, slowed nerve conduction velocity and abnormal F-wave. For all assessments, the patients were kept relaxed, and the skin of the extremities was kept warm (30 °C).

### Laboratory tests

The next morning after enrolment, venous blood samples were obtained from each participant to determine the biochemical indicators. The level of serum insulin was determined using a chemiluminescence method with an immunoassay system (DxI 800, Beckman Coulter), and the serum glucose (using the oxidase method), uric acid (using the uricase-peroxidase method), triglyceride (using colorimetry), total cholesterol (using the cholesterol oxidase method), low-density lipoprotein (LDL) cholesterol (using the selective melt method) and high-density lipoprotein (HDL) cholesterol (using the chemistry modify enzyme method) levels were determined with an automated biochemical instrument (Model 7600, Hitachi). The HbA1c level was determined using ionic exchange HPLC (IE-HPLC) in the D-10 haemoglobin analysis system (Bio-Rad). Homeostasis model assessment (HOMA) was applied to calculate the index of insulin resistance (IR). During the morning, urine samples were also obtained to detect urinary albumin (unit: mg) and creatinine (unit: g), and the urinary albumin-to-creatinine ratio (UACR) was evaluated. The level of urinary albumin was determined using immunoturbidimetry (Immage 800, Beckman Coulter).

### Statistical analyses

Clinical variables are shown for all participants and the two subgroups (those with and without DPN). Normally distributed continuous variables were described as the means ± SDs, while skewed continuous variables were described as the medians (25 and 75% interquartile). Categorical variables were described as frequencies (percentages). To compare the differences in clinical variables between the two subgroups, we used Student’s t-tests, Mann–Whitney U tests or Chi square tests as appropriate.

Both CV-HbA1c and M-HbA1c may be closely associated with DPN. To evaluate the independent impact of CV-HbA1c and M-HbA1c on the risk of DPN, we applied two multivariate logistic regression analysis models to adjust for the other clinical covariates of DPN, and the corresponding odds ratios (ORs) and 95% CI are provided. The first model explored the associations of the second and third tertiles (T2 and T3, respectively) of CV-HbA1c with DPN relative to that of the first tertile (T1). Second, the associations of the T2 and T3 of M-HbA1c with DPN relative to that of the T1 were also assessed. Furthermore, receiver operating characteristic (ROC) analysis was conducted to compare the ability of CV-HbA1c and M-HbA1c to indicate confirmed DPN cases, and the cutoff value of CV-HbA1c to indicate confirmed DPN is provided. Data management and analyses were performed using SPSS statistical software 19.0 (IBM SPSS Statistics). In addition, a value of *p* < 0.05 was considered to be statistically significant.

## Results

### The clinical characteristics of the participants

The clinical parameters of all participants are summarized in Table 1. Among the recruited 563 type 2 diabetic patients, 102 (18.1%) had confirmed DPN. When compared to the patients without DPN, patients with DPN presented with a higher age, diabetic duration, hypertension prevalence, insulin resistance index, initial HbA1c and UACR. However, there were no differences in body mass index, females ratio, systolic/diastolic BP, prevalence of hypoglycaemia, ratio of drinking and smoking, lipid profile or serum uric acid between the patients with and without DPN (*p *> 0.05). Comparisons of hypoglycemic treatments showed that lifestyle intervention and treatment with insulin injection, insulin secretagogues, metformin or thiazolidinediones were similar between the two subgroups (*p *> 0.05). In addition, statins medications were also comparable between the two subgroups. With regard to the glycemic parameters, patients with DPN tended to have a higher M-HbA1c and CV-HbA1c than patients without DPN (*p *< 0.001).

**Table 1:** Clinical characteristics of the participants

Variables	Type 2 diabetes			*t/x* ^*2*^	*p*
	Total	Without DPN	With DPN		
n (%)	563	461 (81.9)	102 (18.1)	–	–
Age (year)	56.4 ± 9.8	56.0 ± 9.9	58.4 ± 9.1	2.254	0.025
Female, n (%)	264 (46.9)	213 (46.2)	51 (50.0)	0.483	0.487
Body mass index (kg/m^2^)	25.4 ± 3.6	25.3 ± 3.7	25.9 ± 3.3	1.533	0.126
Systolic BP (mmHg)	134 ± 17	134 ± 17	137 ± 19	1.623	0.105
Diastolic BP (mmHg)	82 ± 11	82 ± 11	83 ± 10	0.688	0.492
Diabetic duration (year)	5.6 (4.5–8.5)	5.3 (4.4–8.0)	6.7 (4.9–9.6)	–	< 0.001
Hypoglycemic treatments					
Lifestyle intervention alone, n (%)	65 (11.5)	56 (12.1)	9 (8.8)	0.904	0.342
Insulin injections, n (%)	161 (28.6)	127 (27.5)	34 (33.3)	1.369	0.242
Insulin-secretagogues, n (%)	254 (45.1)	212 (46.0)	42 (41.2)	0.781	0.377
Metformin, n (%)	252 (44.8)	199 (43.2)	53 (52.0)	2.612	0.106
Thiazolidinediones, n (%)	294 (52.2)	247 (53.6)	47 (46.1)	1.883	0.170
Hypoglycemia, n (%)	72 (12.8)	58 (12.6)	14 (13.7)	0.098	0.754
Hypertension, n (%)	127 (33.8)	143 (31.0)	46 (45.1)	7.423	0.006
Statins medication, n (%)	209 (37.1)	170 (36.9)	39 (38.2)	0.066	0.797
Smoking, n (%)	144 (25.6)	116 (25.2)	28 (27.5)	0.230	0.632
Drinking, n (%)	116 (20.6)	98 (21.3)	18 (17.6)	0.666	0.415
Triglyceride (mmol/L)	1.74 (1.09–2.79)	1.71 (1.08–2.75)	1.86 (1.19–2.97)	–	0.355
Total cholesterol (mmol/L)	4.85 ± 1.42	4.83 ± 1.32	4.93 ± 1.82	0.646	0.519
HDL cholesterol (mmol/L)	1.06 ± 0.27	1.07 ± 0.27	1.06 ± 0.28	– 0.207	0.836
LDL cholesterol (mmol/L)	2.66 ± 0.83	2.67 ± 0.82	2.60 ± 0.86	– 0.766	0.444
Serum uric acid (μmol/L)	285 ± 98	286 ± 94	277 ± 113	– 0.886	0.376
HOMA-IR	2.86 (2.35–3.49)	2.72 (2.25–3.34)	3.41 (2.89–4.13)	–	< 0.001
Initial HbA1c (%)	8.43 ± 1.15	8.34 ± 1.11	8.81 ± 1.27	3.461	0.001
UACR (mg/g)	20.1 (12.5–36.4)	17.2 (11.1–29.5)	37.2 (25.6–78.8)	–	< 0.001
M-HbA1c (%)	8.85 ± 1.20	8.71 ± 1.16	9.51 ± 1.14	6.309	< 0.001
CV-HbA1c (%)	14.65 ± 3.32	14.22 ± 3.19	16.58 ± 3.20	6.739	< 0.001

*p*-values were determined using Student’s t-tests, Mann–Whitney U tests or Chi square tests as appropriate

### Proportion and ORs of DPN according to CV-HbA1c tertiles

The proportion of participants with DPN increased significantly from 6.9% in the T1 to 19.1% in the T2 and 28.5% in the T3 of CV-HbA1c (*p* for trend < 0.001). Table 2 also shows the ORs of DPN according to the CV-HbA1c tertiles. When compared to the OR of DPN for the participants in the T1 of CV-HbA1c, the ORs for the participants in the T2 and T3 of CV-HbA1c were 3.21 (95% CI 1.64–6.27) and 5.40 (2.83–10.30), respectively. After adjusting for initial HbA1c, M-HbA1c and other clinical risk factors via multiple logistic regression, the corresponding ORs of DPN for the participants in the T2 and T3 versus those in the T1 of CV-HbA1c were 3.61 (1.62–8.04) and 6.48 (2.86–14.72), respectively.

**Table 2:** Proportion and odds ratios (ORs) of DPN according to CV-HbA1c tertiles (95% CI)

	CV-HbA1c tertiles			*p* for trend
	T1 (≤ 12.9%)	T2 (13.0%–15.7%)	T3 (≥ 15.8%)	
n	189	188	186	–
DPN, n (%)	13 (6.9)	36 (19.1)	53 (28.5)	< 0.001
Model 1	1-reference	3.21 (1.64–6.27)	5.40 (2.83–10.30)	< 0.001
Model 2	1-reference	3.11 (1.57–6.16)	5.38 (2.80–10.35)	< 0.001
Model 3	1-reference	3.33 (1.65–6.73)	6.78 (3.33–13.81)	< 0.001
Model 4	1-reference	3.60 (1.64–7.91)	6.35 (2.83–14.19)	< 0.001
Model 5	1-reference	3.61 (1.62–8.04)	6.48 (2.86–14.72)	< 0.001

Model 1: unadjusted model

Model 2: adjusted for age, female ratio, body mass index, systolic/diastolic BP

Model 3: additionally adjusted for diabetic duration, smoking, drinking, statins medications, hypertension and hypoglycaemia

Model 4: additionally adjusted for serum uric acid, lipid profile, HOMA-IR, initial HbA1c, M-HbA1c and UACR

Model 5: additionally adjusted for hypoglycemic treatments

### Proportion and ORs of DPN according to M-HbA1c tertiles

The proportion of participants with DPN increased significantly from 9.6% in the T1 to 17.0% in the T2 and 27.8% in the T3 of M-HbA1c (*p* for trend < 0.001). Table 3 also shows the ORs of DPN according to the M-HbA1c tertiles. When compared to the OR of DPN for the participants in the T1 of M-HbA1c, the ORs for the participants in the T2 and T3 of M-HbA1c were 1.94 (1.05–3.59) and 3.64 (2.03–6.51), respectively. After adjusting for initial HbA1c, CV-HbA1c and other clinical risk factors via multiple logistic regression, the corresponding ORs of DPN for the participants in the T2 and T3 versus those in the T1 of M-HbA1c were 3.63 (1.45–9.09) and 4.05 (1.49–11.01), respectively.

**Table 3:** Proportion and odds ratios (ORs) of DPN according to M-HbA1c tertiles (95% CI)

	M-HbA1c tertiles			*p* for trend
	T1 (≤ 8.42%)	T2 (8.43%–9.33%)	T3 (≥ 9.34%)	
n	188	188	187	–
DPN, n (%)	18 (9.6)	32 (17.0)	52 (27.8)	< 0.001
Model 1	1-reference	1.94 (1.05–3.59)	3.64 (2.03–6.51)	< 0.001
Model 2	1-reference	1.91 (1.02–3.53)	3.55 (1.96–6.40)	< 0.001
Model 3	1-reference	3.77 (1.61–8.84)	5.84 (2.49–13.64)	< 0.001
Model 4	1-reference	3.47 (1.42–8.50)	3.76 (1.41–9.98)	< 0.001
Model 5	1-reference	3.63 (1.45–9.09)	4.05 (1.49–11.01)	< 0.001

Model 1: unadjusted model

Model 2: adjusted for age, female ratio, body mass index, systolic/diastolic BP

Model 3: additionally adjusted for diabetic duration, smoking, drinking, statins medications, hypertension and hypoglycaemia

Model 4: additionally adjusted for serum uric acid, lipid profile, HOMA-IR, initial HbA1c, CV-HbA1c and UACR

Model 5: additionally adjusted for hypoglycemic treatments

### ROC analysis to compare the ability of CV-HbA1c and M-HbA1c values to indicate confirmed DPN

**Fig. 2:**
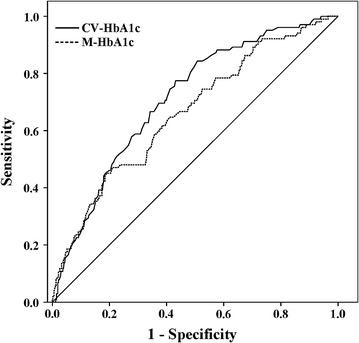
ROC analysis to compare the ability of CV-HbA1c and M-HbA1c to indicate confirmed DPN. AUC of CV-HbA1c and M-HbA1c was 0.711 (95% CI 0.659–0.763) and 0.662 (0.604–0.721), respectively. Optimal cutoff value of CV-HbA1c was 15.15% to indicate DPN; Youden index = 0.324, sensitivity = 66.67% and specificity = 65.73%

ROC analysis was used to compare the ability of CV-HbA1c and M-HbA1c values to indicate confirmed DPN. The area under the curve (AUC) of CV-HbA1c and M-HbA1c was 0.711 (95% CI 0.659–0.763) and 0.662 (0.604–0.721), respectively. CV-HbA1c was better than M-HbA1c in the discrimination between those with and without confirmed DPN. The ROC analysis also showed that the optimal cutoff value of CV-HbA1c to indicate confirmed DPN was 15.15%, with a Youden index of 0.324, sensitivity of 66.67%, and specificity of 65.73% (Fig. 2).

## Discussion

In the present study, we investigated the association of CV-HbA1c with DPN in type 2 diabetic patients. Moreover, we also compared the impact of CV-HbA1c and M-HbA1c on the risk of DPN. The strengths of the study are the following: first, this medium-sized sample of the Chinese population with type 2 diabetes presented with a considerably high prevalence of DPN at 18.1%; second, increased HbA1c variability was shown to be a significant independent contributor to DPN; third, compared with patients in the first CV-HbA1c tertile, those in the second and third CV-HbA1c tertiles were associated with an increased risk for DPN, with multiple-adjusted ORs of 3.61 (1.62–8.04) and 6.48 (2.86–14.72), respectively; fourth, the ability of CV-HbA1c to indicate confirmed DPN was superior to that of M-HbA1c; fifth, the optimal cutoff value of CV-HbA1c to indicate DPN was 15.15%, and its corresponding sensitivity and specificity were 66.67% and 65.73%, respectively.

### Glycemic variability and diabetic complications

The short-term glycemic variability index, especially the mean amplitude of glycemic excursions (MAGE) from continuous glucose monitoring, is indicative of a more adverse effect on the pathogenesis of diabetic vascular complications than indicators of mean hyperglycaemia [8, 13]. However, the relationship between short-term glycemic variability assessed by MAGE and the presence of macrovascular and microvascular complications are still controversial. Su et al. [14] found that MAGE was associated with the presence and severity of coronary artery disease in type 2 diabetes, and Xu et al. [15] revealed that MAGE was a significant indicator for detecting CAN in newly diagnosed type 2 diabetes. However, Caprnda et al. [16] failed to show an association of MAGE with micro- and macrovascular complications in type 2 diabetes. This discrepancy may be because short-term glycemic variability is not sufficient to explain the diabetic vascular complications, which are characterized by a chronic course. Therefore, long-term glycemic variability may be a reliable predicator for vascular complications in diabetes. Long-term glycemic variability commonly refers to the glycemic variability over several months or years, which is usually assessed by the annual variability of fasting plasma glucose (FPG) levels, glycated albumin (GA) or HbA1c. Annual FPG variability was found to be a significant risk factor for renal disease [17], ischaemic stroke [18], Alzheimer disease [19], occurrence of cancers [20], and all-cause and cardiovascular mortality [21] in type 2 diabetic patients, as well as hip fracture in older type 2 diabetic patients [22]. Moreover, a higher HbA1c variability was associated with a higher risk of microalbuminuria [23], diabetic retinopathy, adverse cardiovascular events and mortality [24] in type 2 diabetic patients. With respect to diabetic neuropathy, two studies by Jun et al. [10, 25] have shown that both annual GA variability and HbA1c variability contribute to CAN in type 2 diabetic patients. Although our previous study showed that short-term glycemic variability assessed by MAGE was associated with DPN in a small sample of type 2 diabetic patients with well-controlled average glucose levels [26], the association between short-term glycemic variability and DPN is still under debate [27]. Short-term glycemic variability may not be sufficient to explain the occurrence of DPN, and long-term glycemic variability may play an important role in the development of DPN. A recent study by Yang et al. [28] revealed that annual FPG variability was a potent predictor for DPN in type 2 diabetes. In the present study, we found that increased HbA1c variability evaluated by CV-HbA1c is a significant independent contributor to DPN, which adds to the evidence that long-term glycemic variability is associated with a high risk of DPN in type 2 diabetic patients.

### Potential risk factors and DPN

The incidence of DPN is based on the incidence of diabetes, and hyperglycaemia and coexisting metabolic risk factors may promote DPN. Previous studies identified clinical parameters, such as ageing, long duration of diabetes [29], high HbA1c and GA [30], raised body mass index, hypertension, dyslipidaemia [31], insulin resistance [32], low serum total bilirubin [33], elevated serum cystatin C [34], C-peptide and vitamin D deficiency [35, 36], high thyroid-stimulating hormone [37], increased urinary albumin and decreased glomerular filtration rate (GFR) [38], anaemia [39] and arterial stiffness [40], as potential risk factors for DPN. Moreover, inflammatory markers, i.e., white blood cell parameters [41], tumour necrosis factor-α [42], serum C-reactive protein (CRP) [42], etc., have been observed to be related to diabetic neuropathy. Furthermore, endoplasmic reticulum stress also plays a vital role in the development of DPN [43]. In the present study, in addition to long-term hyperglycaemia assessed by M-HbA1c, long-term glycemic variability assessed by CV-HbA1c was observed to be independently associated with DPN in type 2 diabetic patients. Furthermore, the ability of CV-HbA1c to indicate DPN was superior to that of M-HbA1c. We also used ROC analysis to determine that the optimal cutoff value of CV-HbA1c to indicate DPN was 15.15%.

Additionally, our present study also showed that patients with DPN had a higher age, diabetic duration, hypertension prevalence, insulin resistance index (HOMA-IR) and UACR than patients without DPN, in agreement with previous findings [29, 31, 32, 38]. These risk factors are modifiable, except for age and diabetic duration. Insulin resistance, the basis of the pathophysiology of type 2 diabetes, is also a key factor underlying DPN [5, 32]. Insulin resistance causes impaired insulin signalling, primarily leading to inhibition of the phosphoinositide 3-kinase (PI3K)/Akt signalling pathway, which in turn results in injury to the nervous systems. UACR, a potent indicator for diabetic nephropathy, is closely associated with DPN [44]. DPN may be driven or accompanied by diabetic nephropathy. Moreover, diabetic vascular complications do not always occur in isolation but are often found as a group in patients. Mohammedi et al. [45] demonstrated that the presence of microvascular (including peripheral neuropathy) or macrovascular disease at baseline is independently associated with an increased risk of major clinical micro- and macrovascular events and death in patients with type 2 diabetes. Khandoker et al. [46] revealed that peripheral neuropathy and other microvascular complications could affect heart rate variability. The previous study and our present results imply that DPN and other diabetic vascular complications are interconnected. Therefore, multi-approach targeting of HbA1c variability and other modifiable risk factors may improve DPN and its accompanying vascular complications in type 2 diabetic patients.

Unlike our study, some previous studies have reported associations between hypoglycemic treatments and DPN. Pop-Busui et al. [47] revealed that the occurrence of DPN in patients given insulin injections was more than in those taking insulin-sensitizing agents. Katulanda et al. [48] found that sulphonylureas treatment was an important risk factor for DPN. The reason for this discrepancy may be that the doses of the hypoglycemic agents and combination therapy used by the patients in our study were different from those in the previous studies.

### Possible mechanisms linking HbA1c variability and DPN

Long-term glycemic variability, as assessed by HbA1c variability, may promote oxidative stress [49], which in turn may mediate tissue and cell damage through four main molecular pathways, including augmented flux through the polyol pathway, overproduction of precursors of advanced glycation end products, overactivation of protein kinase C isoforms and enhanced activity of the hexosamine pathway [50]. Moreover, HbA1c variability may also enhance expression of a marker of systemic inflammation [49], which is linked to vascular damage. Another important mechanism by which HbA1c variability participates in diabetic complications is through cellular metabolic memory, which may differ from short-term glycemic variability [9]. Prolonged exposure to glycemic variability produces a detrimental condition involving excessive cellular markers of DNA damage and hyperactivation of tumour suppressor transcription factor p53, which may lead to a greater metabolic memory effect than exposure to sustained hyperglycaemia [51]. These cell damages can occur in neurons and supporting tissue, including neuroglial cells and capillaries, all of which may result in nervous dysfunction and neuropathy [5]. Therefore, HbA1c variability may be a potential factor associated with DPN risk.

## Limitations

Our study has certain limitations that must be addressed. First, although a positive association between HbA1c variability and the presence of DPN was found in this cross-sectional observational study, whether the association is causal is uncertain. A prospective study is required to compensate for this weakness. Second, the present study was performed in a Chinese population with type 2 diabetes, and the generalizability of our results should be assessed. Third, we did not investigate HbA1c variability in relation to indices of oxidative stress, inflammation or endothelial dysfunction. Fourth, a 1-year period for the evaluation of HbA1c variability is a relative short period of time when compared to that used in some previous studies [10, 25]. Fifth, we did not evaluate the relationship between HbA1c variability and DPN severity.

## Conclusions

In summary, increased HbA1c variability is closely associated with DPN in type 2 diabetic patients and could be considered a potent indicator for DPN in these patients. In addition, clinical strategies targeting HbA1c variability may provide therapeutic methods to ameliorate DPN in these patients.

## Abbreviations

DPN: diabetic peripheral neuropathy
BP: blood pressure
LDL: low-density lipoprotein
HDL: high-density lipoprotein
UACR: urinary albumin-to-creatinine ratio
HOMA: homeostasis model assessment
IR: insulin resistance
HbA1c: glycosylated haemoglobin A1c
CV-HbA1c: coefficient of variation of four HbA1c values over the year preceding enrolment
M-HbA1c: mean of four HbA1c values over the year preceding enrolment
ROC: receiver operating characteristic
T1: first tertile
T2: second tertile
T3: third tertile
CAN: cardiovascular autonomic neuropathy
FPG: fasting plasma glucose
MAGE: mean amplitude of glycemic excursions
GA: glycated albumin
